# Evidence of active transport involvement in morphine transport via MDCKII and MDCK-PGP cell lines

**Published:** 2010

**Authors:** S.O. Mashayekhi, M.R. Sattari, P.A. Routledge

**Affiliations:** 1 *National Public Health Management Research Center, Faculty of Pharmacy, Tabriz University of Medical Sciences, Tabriz, I.R.Iran*; 2 *Hematology and Oncology Research Center, Faculty of Pharmacy, Tabriz University of Medical Sciences, Tabriz, I.R.Iran*; 3 Department of Pharmacology, Therapeutics and Toxicology, Landough Hospital, College of Medicine, Cardiff University, UK

**Keywords:** Morphine transport, P-glycoprotein, Probenecid-sensitive transport, Multidrug resistance related protein

## Abstract

Several transporters appear to be important in transporting various drugs. Many patients, who receive morphine as analgesic medication, also receive other medications with potency of changing morphine transport by affecting P-glycoprotein (P-GP) and oatp2 transport system. This could influence morphine pharmacokinetics and pharmacodynamics. The aim of present study was to elucidate the transport mechanisms involved in transporting morphine via MDCKII and MDCK-PGP cells. Morphine permeability was examined in the presence of various compounds with ability in inhibiting different transport systems including: digoxin, probenecid and d- glucose. The effect of morphine concentration changes on its transport was also examined. Morphine concentration was measured using HPLC with electrochemical detector. Morphine permeability via a MDCK II cells was greater than sucrose permeability, and reduced when a P-GP expressed cell line was used. Its permeability was increased significantly in the presence of a strong P-GP inhibitor. Morphine permeability decreased significantly in the presence of digoxin but not in the presence of d-glucose or probenecid. These results showed that morphine was a P-GP substrate, and digoxin related transporters such as oatp2 were involved in its transport. Morphine was not substrate for glucose or probenecid-sensitive transporters.

## INTRODUCTION

At first, it was thought that blood-borne substances entrance to brain fluids happens only by free diffusion which depend on their lipid partition coefficient(1) and molecular size(2). But Oldendrof and colleagues (1972) suggested that there were specialised transport systems mediating the influx and/or efflux of many substances(3). Several transporters such as P-glycoprotein (P-GP), probenecid-sensitive transport mechanism, multidrug resistance related protein 1-3 (Mrp 1-3), the organic anion transporter family (Oat 1-3), and the organic anion transporter polypeptide family (Oatp 1-3) appear to be important in transporting various drugs in and out of blood tissue barrier(4). P-GP is located in tumour cells and also in normal tissues such as the liver, kidney, intestine, and brain(5),(6)and appears to be an important functional component of the blood brain barrier (BBB). It acts as an efflux pump and removes substrate from the interior cells against their concentration gradients. Clinically important interaction between morphine and P-GP substrates is shown when anticancer drugs such as taxanes, vinca alkaloids and platinum salts produce neuropathy(7). The pain caused by neuropathies is difficult to be managed even with opioids(7,8). Campa and co-workers showed that polymorphism of P-GP was related to morphine efficiency in the treatment of severe pain(9).

Morphine is the golden drug in the treatment of moderate to severe pain(10). The pharma-cological responses to morphine are well related to the CNS disposition of morphine(11). Morphine passage into the BBB is well recognized but compared to many drugs and other opioids, its penetration rate is rather limited(3).

There is some evidence that morphine could be a P-GP substrate. Therefore, P-GP could play an important role in morphine activity and also its toxicity by increasing uptake at the level of the BBB and also by decreasing renal elimination. The presence of P-GP inhibitors might alter morphine pharmacokinetics and/or pharmacodynamics. On the other hand, it is also possible that P-GP is not the only mechanism for transporting morphine and other transporters could be effective in its transport via various barriers such as BBB.

This study was designed to evaluate the role of P-GP and other transporters in the transport of morphine across the cell monolayer using transwells containing a monolayer of MDCK II or MDR-PGP cells.

## MATERIALS AND METHODS

### 

#### Material and cells

Morphine, probenecid, digoxin and cyclosporin (Sigma-Aldrich, UK), d-glucose (BDH Chemicals Ltd, England),[6, 6’(n)- ^3^H] sucrose (Amersham Bioscience AB), Dulbecco’s modified eagle’s medium with high d-glucose [DMEM, Gibco-BRL], penicillin and streptomycin (Invitrogen, UK), trypsine (Invitrogen, UK), foetal bovine serum (FBS, Invitrogen, UK), and organic liquid: Optiphase ‘Hisafe’3 (Wallas, Perkin Elmer. Loughborough, Leics, England). Hanks’ balanced salt solution (Hank’s J. 1976) and phosphate buffer solution (PBS) were made in the lab using analytical grade chemicals. Polycarbonate membrane Transwell ^®^(6.5 mm diameter, 0.4 μ m pore size, tissue culture treated, polystyrene plates, 12/plate, 48/case, sterile, Costar^®^, Corning Incorporated, USA) was used for transport studies.

Culture media was consisted of 10% FBS, 2% penicillin/streptomycin and 88% DMEM mix in a sterile plastic tube and kept at 2 to 8 °C for maximum of 7 days.

Madin-Darby Canine Kidney epithelial cell lines (MDCK-II, passages 3-7), and MDCK I-MDR (MDCK-PGP or MDCK WT, passages)(6–10) were purchased from Borst- Amsterdam. MDCK-PGP is a MDCK-II recombinant clone containing the human MDR-I PGP gene.

Morphine was dissolved in a solution of 10% v/v ethanol/water as stock solution. This stock solution was diluted to reach required concentration(s) for each experiment with working buffer whilst ethanol concentration was kept constant (10%) for each dilution. Morphine concentration was measured in samples using high-performance liquid chromatography with electrochemical detector. The method of measurement of morphine was sensitive, specific and able to detect small changes in morphine concentrations(12).

When cell growth and cell number were sufficient, the cells were transferred into the transwells. The number of cells in each well was calculated using Eq. 1

Eq. 1 $$\mathrm{Number}\;\mathrm{required}\;\mathrm{cells}/\mathrm{Insert}\;=\;40,000\;\mathrm{cells}/{\mathrm{cm}}^{2}\;\times \;\mathrm{Area}\;\mathrm{of}\;\mathrm{insert}$$

In 48/case plates, with area of 0.3318 Cm^2^for each insert, the required cells per insert were 13,272 cells. This number of cells was dispensed in 0.25 ml media for each insert and the cells suspension was poured into apical chambers of the inserts. Each treatment group comprised 4 replicates. One ml of cell free media was added to basal chambers. One insert without any cell but containing media was prepared in order to measure background resistance when measuring transepithelial electric resistance (TEER) of inserts containing monolayer of cells. The plates were kept in an incubator at 37 °C in 5% CO_2_and the media from both apical and basal chambers were changed every 2 days. The transport experiment was carried out on fifth or sixth day after plating.

#### Transport study

After completing the incubation period, TEER of each Transwell inserts was measured using an EVOM epithelial voltammeter (WIP, Sarasota, FL) and the Transwell inserts were distributed evenly between treatment groups, based on the TEER measurement. Then the media from inserts (both apical and basal chambers) were replaced with the working buffer for an equilibratin period of 30 min prior to the start of the transport study. The transport study in the direction of apical to basal (A→B) was initiated by replacing 0.25 ml of apical solution with the same volume of morphine solution in the working buffer (T = 0 min). A sample (0.2 ml) was removed at each interval from the basal chamber and replaced with the same amount of the working buffer over a period of 100 min (after 15, 30, 45, 60, 80 and 100 min). The plates were stirred on an orbital shaker at a rate of 125 rpm at 37 °C. At final sampling, a sample was also removed from the apical chamber as well as the basal chamber in order to examine any possible glucorinidation of morphine by cells. The corrected cumulative concentrations of drug were determined based on the amount of removed and added buffer in each series of wells at each interval and the corrected concentrations were used for calculating the apparent permeability coefficient (ρ) according to the Eq. 2:

Eq. 2 $$\mathrm{dM}/\mathrm{dt}=\rho \;\times \;A\;\times \;C_{0}$$

where dM/dt is the rate of change in cumulative mass of morphine transferred to the receiver chamber, A represents the surface area of Transwell membrane and C0 represents the initial concentration of substance in the donor chamber assumed to remain essentially constant (i.e., <5% loss) throughout the experiment. Hanks’ balanced salt solution was used for the experiments.

#### Sucrose transport via MDCK II and MDR-PGP cells

The transport of sucrose as an extracellular marker(13) was compared with morphine transport via MDCK II and MDR-PGP cells. Radiolabel sucrose (8,000,000 DPM/ml) in DMEM replaced buffer in apical chambers. Sampling was performed by removing 0.2 ml from the basal chambers and replacing the same volume with DMEM at 15, 30, 45, 60, 80 and 100 min time intervals.

#### Effects of morphine concentrations on its transport using MDCK II cells

Four concentrations of morphine (61.8, 100, 200 and 400 μg/ml) was prepared in Hank’s balanced salt solution and content of apical chambers were replaced by 0.25 ml of these solutions (Time = 0 min). The usual procedure was followed.

#### Identification of morphine as a PGP substrate

Two methods were used to show that morphine is a PGP substrate. The first method was comparing its transport via MDCK II and MDR-PGP; MDCK II cells with expressed P-GP cells(14), and the second method was addition of a known potent PGP inhibitor, cyclosporin (10 μM)(15), and assessing its effect on morphine transport.

#### Effects of other transporter systems on morphine transport via MDCK II cells

The effects of probenecid (10 μM), d--glucose (5 μM), and digoxin (5 μM) on morphine transport was examined in separate experiments. These drugs were dissolved in Hank’s buffer separately and used as working buffer in related experiments. Morphine solution (61.82 μg/ml) in the working buffer was also made. In separate experiments the buffer in the apical and basal chambers were removed and replaced with related Hank’s buffer solution 30 min prior to the experiments. Then the related morphine solution was replaced with the buffer in the apical chambers. As before, samples were removed from basal chamber at different intervals and replaced with related morphine free working buffer. Each treatment group comprised 4 replicates. In similar experiment morphine (6.182 μg/ml) permeability in the presence morphine 6 glucoronide (M6G, 100 μg/ml) was also examined.

As control experiment, 4 Transwell inserts were prepared and usual procedure was followed using morphine solution in Hank’s buffer without any other extra drug for replacing the apical chamber and drug free Hank’s buffer for replacing the removed samples from basal chamber.

## RESULTS

### 

#### Sucrose transport via MDCK II and MDR-PGP cells

In the present study morphine was shown to be able to cross a layer of MDCK II and MDR-PGP cells more than sucrose could. Sucrose permeability was significantly lower than morphine permeability (*P*<0.001). Morphine permeability was 60 and 50 times higher than sucrose permeability via MDCK II and MDR-PGP cells, respectively (Table 1).

**Table 1:** The permeability level of morphine and sucrose via MDCK II cells and MDR-PGP cells.

		Permeability (×10^6^ cm/s)	

Substance (concentration)	MDCK II cells	MDR-PGP cells	Mean differences, R^2^, P value, N
Morphine	27.86 ± 1.22	18.67 ± 0.11	9.1, R^2^ = 0.98, P=0.0008, N=4
Sucrose (8,000,000 DPM/ml)	0.46 ± 0.09	0.38 ± 0.04	0.08, R^2^ = 0.26, P>0.01, N=4

#### Effects of morphine concentrations on its transport using MDCK II cells

The A → B transport study of morphine showed that its permeability via MDCK II monolayer cells increased significantly when its concentration increased from 61.82 to 400.0 μg/ml (R^2^= 0.971, *P* = 0.015). The results are shown in Table 2.

**Table 2:** The permeability level of different concentrations of morphine in the presence of MDCK II.

Morphine Concentration* (μg/ml)	Permeability ± SD (10^6^ cm/s)
61.82	27.86 ± 1.22
100.0	30.88 ± 1.34
200.0	42.94 ± 4.57
400.0	54.60 ± 1.77

* Hank’s buffer was as working buffer

#### Identification of morphine as a PGP substrate

Comparing morphine permeability via two cell lines showed that morphine permeability via MDR-PGP cells was significantly lower than its permeability via MDCK II cells (18.67 ± 0.11×10^6^cm/s compare to 27.86 ± 1.22×10^6^cm/s, P<0.001).

**Fig. 1:**
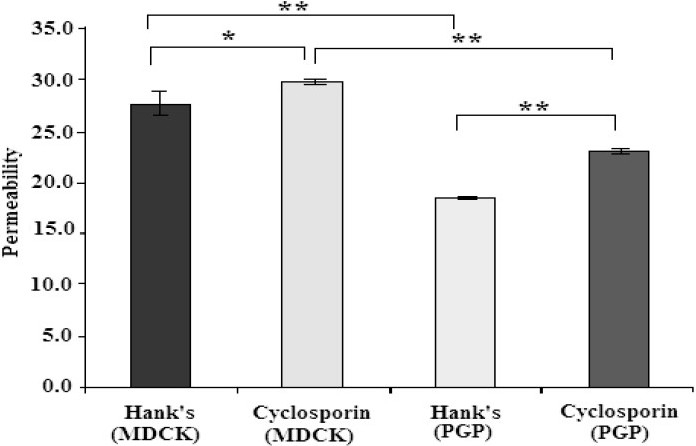
Effects of the presence of cyclosporin on morphine permeability via two cell lines (*P<0.01, **<0.001).

Cyclosporin as a potent PGP inhibitor(15)competitively inhibits PGP activity(16)and reduces its efflux activity and therefore increases the transport of PGP substrates. Addition of cyclosporin caused a significant increase in morphine permeability via MDCK II cells compared to cyclosporin absence (from 27.86 ± 1.22 to 29.64 ± 0.21×10^6^cm/s, Fig. 1). Furthermore, when cyclosporin was added to MDR-PGP cells, morphine permeability increased to 22.95 ± 0.21×10^6^cm/s which was significantly higher than morphine permeability via MDR-PGP cells in the absence of cyclosporin (*P*<0.001).

#### Effects of other transporter systems on morphine transport via MDCK II cells

**Fig. 2:**
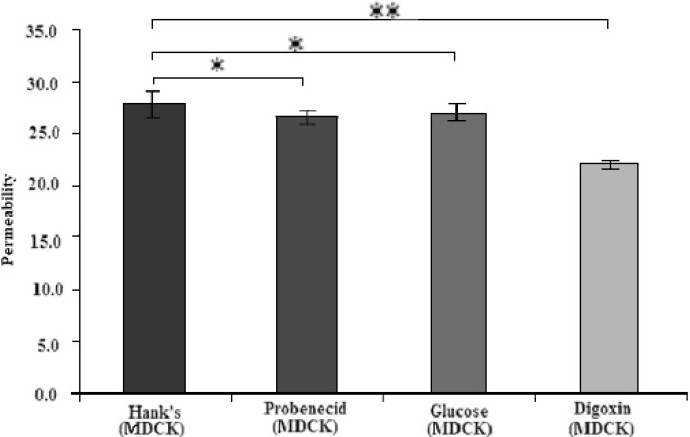
Effects of the presence of various transporters substances on morphine permeability via MDCKII cell lines (*P<0.05, **P<0.001).

**Fig. 3:**
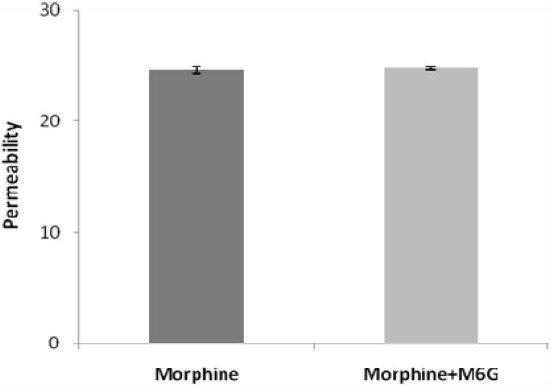
Effect of M6G on morphine permeability via MDCKII cell lines.

In our study probenecid, d-glucose or M6G had no significant effects on morphine permeability (*P*>0.05), but digoxin caused a significant fall in morphine transport (*P*<0.001). The results are shown in Table 3 and Fig. 2 and 3. Finally glucorinidation of morphine was not detected during transport study.

**Table 3:** The permeability (±SD) of morphine in the presence of various drugs.

	Hank’s	Probencid	Glucose	Digoxin	M6G
Permeability (×106 cm/s)	27.72 ± 1.22	26.60 ± 0.76	27.01 ± 0.83	24.23 ± 0.39	24.73 ± 0.17
Number of cells	4	4	4	4	4

## DISCUSSION

In our study, for sucrose and morphine, the amount of transepithelial movement in the direction of apical to basal was 0.1%, and 6%, respectively after incubation for 100 min. Morphine permeability was 60 times higher than sucrose. These experiments confirmed that morphine could cross a layer of MDCK II cells more rapidly than sucrose. In another study, the differences between morphine and sucrose partition coefficient was even greater, with morphine more able to cross the membrane (340-fold higher)(13). On the other hand, Wandel and co-workers (2002) showed that less than 1% of morphine could cross the membrane(17). They observed a lower membrane transport for morphine compared with the present study and Huwyler and colleagues study. The differences in the used cell lines, which might contain different types and numbers of transporters on their apical and basal side, may be an explanation for the differences between the results of these studies.

Examining morphine transport at a range of concentrations showed its permeability increased significantly (2-fold) with an increase in its concentration (6.5-fold). The results related to morphine was similar to results of another study(18). This could mean that increased morphine concentrations might have saturated PGP, an efflux transporter.

In order to identify a drug as PGP substrate, two methods can be applied: one is to compare results of two series of cell lines, with and without expressed PGP such as MDCK II and MDR-PGP cells which is MDCK-II recom-binant clone containing the human MDR-I PGP gene. Another way is to compare its transport in the presence and absence of a known PGP inhibitor such as cyclosporin A, amiodarone, quinacrine, verapamil, quinidine, vinblastine, vincristine, chloroquine, colchi-cines, etopside, doxorubicine(15). In the present study, MDR-PGP cells were significantly less permeable to morphine than were MDCK II cells. This suggests that morphine is a P-GP substrate. Using different cell lines and despite reporting a low level of morphine permeability, Wandel and colleag ues (2002) also reached the same conclusion(17). Cyclosporin is a potent P-GP inhibitor,(15) and inhibits P-GP activity, reduces its efflux activity, and therefore increases the transport of P-GP substrates. The present study indicated that morphine permeability increased significantly in the presence of cyclosporin, further indication that morphine is a P-GP substrate. The change in permeability was greater in the presence of MDR-PGP cells than MDCK II cells, an expected finding because of the higher concentrations of P-GP on MDR-PGP cells. Previously it was shown that GF120918, another potent PGP inhibitor, reduced morphine brain distribution by 42% in rats(19). By showing that morphine is a P-GP substrate using kidney cell lines, it is highly possible that its transport could be effected in the presence of P-GP inhibitors, wherever P-GP presents. The examples of such places are renal tubule and BBB, and therefore it is possible to observe clinically important interaction when morphine with P-GP inhibitors are co-prescribed, as mentioned earlier.

It has been shown that probenecid enhances CNS uptake of morphine 3- glucoronide (M3G) by an increase in its influx clearance into the brain of rats(20)and because of the structural similarity of morphine to M3G, the consequences of probenecid co-addition with morphine was investigated, however, we failed to show a significant effect on morphine permeability caused by probenside. This could mean the glucoronide section of M3G might be the important section in attaching the molecule to the transporters.

Morphine permeability decreased significantly in the presence of digoxin. If digoxin worked only as a P-GP inhibitor, thus an efflux pump with luminal location(21), an increase in morphine permeability should have been seen, similar to cyclosporin effect. It is therefore possible that digoxin reduced morphine permeability via mechanism(s) other than P-GP inhibition. These findings indicates that morphine is a substrate to the same transporters as digoxin, and since digoxin was shown to be transported by both P-GP and oatp2(22), the latter transporter could be the one affected our results. Oatp2 transporters are placed at both the luminal and basolateral sides of the brain epithelial cell and unlike P-GP act both on influx and efflux(23). It is possible that digoxin could have a significant effect on morphine uptake by brain which might be clinically relevant.

In mammals d-glucose is rapidly transported by glucose transporters family (GLUT) and the main glucose transporters in foetal tissue and tissue culture cells is GLUT1 which is also expressed at the highest level in the cells of blood tissue barriers like the BBB and kidneys(24). Polt and co-workers(25)showed that glycopeptide enkephalin analogues produces analgesia in mice, unlike most peptides which are unable to cross the BBB and reach the receptors in order to produce a response. They suggested that glycopeptide analogues crossed the BBB via the same transporters as d-glucose itself. It seems that morphine does not possess such a power and this study showed that addition of d-glucose did not have any significant effect on morphine permeability.

In these experiments, it was shown that M6G did not have an effect on morphine permeability. It is likely that M6G does not have P-GP inhibitory properties. Unlike Aasmundstad and colleagues study, which showed the biotransformation of morphine in freshly isolated parenchymal and non-parenchymal liver cells from rats and guinea pigs in a suspension culture(26), the present experiment failed to show any detectable glucuronidation of morphine into M6G in the employed system.

Morphine is an important agent that is prescribed in a wide range of disease and co-administrated with many other drugs including the anti-cancer drugs(27). We previously showed that some of anti-cancer medications interfere on morphine effects by affecting its protein binding(28) but additional to this method of interaction, these co-prescribed medications which are inhibitors of P-GP or other transporters inhibitor could interfere on morphine transport in and out of brain or renal tubes. It means that morphine, as a substrate for various transporters, could be involved in drug-drug interactions. The effects may be an increase or reduction in morphine brain uptake or its elimination and therefore affect its analgesia properties, an important clinical issue.

## CONCLUSION

The results of the present study could be the explanation of the inter and intra-patient variation seen among those who receive morphine as pain reliever. Further experiments are needed to examine the extension of these effects in the clinical settings.
